# Revealing the pathogenesis of autoimmune hepatitis and research progress in drug discovery from hepatic immune cells and intercellular communication signaling mechanisms

**DOI:** 10.3389/fimmu.2026.1871133

**Published:** 2026-06-18

**Authors:** Jie Yang, Hongying Zhou, Xiao Ma, Xuelin Zhou, Shizhang Wei, Yanling Zhao, Chunyu Li

**Affiliations:** 1 Department of Pharmacy, the First Affiliated Hospital of China Medical University, Shenyang, Liaoning, China; 2 School of Pharmacy, Chengdu University of Traditional Chinese Medicine, Chengdu, Sichuan, China; 3 Department of Pharmacology, School of Basic Medical Sciences, Capital Medical University, Beijing, China; 4 Department of Pharmacy, National Cancer Center/National Clinical Research Center for Cancer/Cancer Hospital, Chinese Academy of Medical Sciences and Peking Union Medical College, Beijing, China; 5 Department of Pharmacy, Medical Security Center, Chinese PLA General Hospital, Beijing, China

**Keywords:** autoimmune hepatitis, cytokine network, drug discovery, immune cells, intercellular communication

## Abstract

Autoimmune hepatitis (AIH) is an immune-mediated chronic liver disease with an increasing incidence. The complex pathogenesis of AIH poses significant challenges for clinical treatment. In recent years, with the introduction of cutting-edge concepts such as “immune–metabolic interplay” and “intercellular communication networks,” along with novel detection methods and technologies, research on the pathogenesis of AIH has shifted from a single-molecular-mechanism perspective to a systematic regulatory network analysis. This shift has not only provided more research evidence for a deeper understanding of the molecular biological mechanisms underlying AIH but also offers a potential reference for the development of targeted therapeutic drugs for AIH based on novel targets. Therefore, this review starts with aberrant activation signals from key immune cells—including dendritic cells (DCs), macrophages (Mφ), and T/B cells—and integrates the intercellular signaling communication mechanisms between hepatocytes, cholangiocytes, and immune cells to systematically summarize the key molecular biological mechanisms and targets identified in recent years, providing a reference for future elucidation of the critical mechanisms of AIH. On this basis, the review further integrates the current application and research progress of clinically used AIH therapeutic drugs, as well as those at various stages of development, including potential therapeutic compounds. It discusses the limitations of current clinical drugs and evaluates the feasibility and future application potential of potential compounds for AIH treatment in preclinical and clinical studies, thereby offering comprehensive research evidence for the management of AIH.

## 1 Introduction

AIH is a chronic, progressive inflammatory liver disease caused by an immune-mediated attack that specifically targets hepatocytes. Its typical pathological features include interface hepatitis, elevated serum transaminase levels, hypergammaglobulinemia, and autoantibody positivity (1). Persistent progression can gradually evolve into cirrhosis and end-stage liver failure (2). The clinical manifestations of AIH are highly heterogeneous and non-specific, mainly comprising three types: the most common is the insidious-onset chronic form, where patients may have no obvious symptoms or only non-specific manifestations such as fatigue and arthralgia; the second is the acute-onset type, whose clinical presentation resembles acute viral hepatitis, with marked jaundice, fatigue, nausea, and other symptoms, making misdiagnosis likely; a small number of patients may present with fulminant liver failure, characterized by acute onset, coagulation dysfunction, and hepatic encephalopathy, representing a critical condition (3). In addition, some cases may present as acute-on-chronic liver failure (ACLF), which progresses rapidly, has a high short-term mortality rate, and is a major cause of death in patients with chronic liver disease (4). This diverse clinical presentation poses significant challenges for the timely diagnosis and treatment of AIH. The pathogenesis of AIH is not yet fully understood; genetic factors, immune factors, and environmental factors are currently considered to be the predisposing factors. These factors can induce abnormal T-cell immune responses against liver antigens, triggering the characteristic histological changes of AIH, including hepatic necroinflammation and liver fibrosis (5). In AIH, imbalances in immune cells such as regulatory T cells (Tregs) and effector T cells (Teffs) are involved in the initiation and persistence of liver injury (6). Studies have revealed that the occurrence and development of AIH are far from being a single cellular or molecular event; rather, they constitute a dynamic process co-driven by intricate intracellular signaling networks and complex intercellular communication systems. The core mechanism includes a functional imbalance between effector immune cells (e.g., Th17 cells, Th1 cells, and CD8+ tissue-resident memory T cells) and Tregs (7–9), which leads to disruption of immune tolerance and promotes autoimmune liver injury (10).

Current traditional treatments for AIH are based on corticosteroids (e.g., prednisolone or budesonide), often in combination with immunosuppressants such as azathioprine for induction and maintenance therapy, with some patients requiring long-term medication to prevent relapse (11, 12). However, corticosteroid-related side effects (e.g., diabetes, osteoporosis) significantly affect patient compliance and quality of life (13–15); According to clinical studies from Europe and North America, approximately 10%-20% of AIH patients show poor response to first-line immunosuppressive therapy or develop refractory AIH (16); it should be noted that the generalizability of this proportion across different geographic populations requires further validation (17); the evidence base for long-term immunosuppressive therapy is weak, and the risk of relapse after drug withdrawal is high (18). With a deepening understanding of AIH pathogenesis, research on therapeutic agents is shifting from traditional broad-spectrum immunosuppression toward precision immune regulation. At the intersection of innate and adaptive immunity, CD20 inhibitors targeting B-cell depletion (e.g., rituximab) and BAFF/BLyS inhibitors regulating plasma cell activation have shown significant clinical efficacy; however, CD20 inhibitors carry risks of infection and hepatitis B reactivation in susceptible patients, while BAFF/BLyS inhibitors lack large-scale clinical trial data for the treatment of AIH (19, 20). In the field of T-cell function regulation, low-dose IL-2 therapy, which can restore Treg function, has demonstrated potential; nevertheless, although low-dose IL-2 significantly and sustainably increases circulating Tregs, it fails to promote the accumulation of intrahepatic regulatory T cells (21, 22). Furthermore, inhibitors of the PDGF-βR and TGF-β pathways targeting hepatic stellate cell (HSC) activation (23, 24), as well as emerging epigenetic targets such as HDAC and BRD4 (25, 26), are showing potential for reversing fibrosis in preclinical studies. Together, these targets constitute a multi-level therapeutic strategy ranging from controling inflammatory responses to re-establishing immune tolerance.

This review used PubMed as the literature database, with the search time range limited to January 2016 to March 2026 (the last 10 years). Search terms combined MeSH terms and free words, including: “autoimmune hepatitis”, “AIH”, “immune cells”, “dendritic cells”, “macrophages”, “T cells”, “B cells”, “cross-talk”, “signaling pathways”, and “drug discovery”. The majority of the cited articles were published in the last 10 years (2016–2026); a limited number of earlier classic publications (before 2016) were supplementarily included via manual searching and reference tracking only when they provided critical background or mechanistic foundations. Inclusion criteria were: (1) original research articles, clinical trials, or authoritative reviews addressing AIH pathogenesis, immune cell function, intercellular communication, or therapeutic interventions (both clinical and preclinical); (2) articles written in English. Exclusion criteria were: (1) non-peer-reviewed items (e.g., conference abstracts, editorials, commentaries); (2) studies focusing on other liver diseases without AIH-specific data; (3) *in vitro* studies lacking *in vivo* or clinical relevance.

This review aims to systematically outline the aberrantly activated core intracellular signaling mechanisms in AIH, followed by an in-depth analysis of the key intercellular interaction networks within the liver microenvironment, thereby providing a reference for future elucidation of the critical mechanisms underlying AIH. Furthermore, it integrates and synthesizes the application and research progress of clinically used therapeutic agents, as well as drug candidates and potential therapeutic compounds at various stages of development for AIH, offering comprehensive evidence for a deeper understanding of the current status of AIH treatment.

## 2 Role of autoantigen presentation signals in AIH

As a highly immune-tolerant organ, the liver is continuously exposed to a variety of foreign and endogenous antigens and is capable of effectively clearing pathogens while maintaining immune tolerance to harmless antigens (27). This balance depends on a complex antigen presentation system, including DCs, B cells, Kupffer cells (KCs) and other conventional antigen-presenting cells (APCs), as well as liver sinusoidal endothelial cells (LSECs), HSCs, hepatocytes and other unconventional APCs (28). In AIH, hepatic autoantigens are presented by APCs to undifferentiated Th0 lymphocytes (i.e., the transitional state of naïve CD4^+^ T cells after antigen stimulation but before lineage commitment, with their differentiation direction dictated by the cytokine milieu), triggering a cascade of effector immune responses that ultimately lead to the release of inflammatory cytokines, resulting in hepatocyte injury and interface hepatitis (29, 30). Dysfunction of this system leads to loss of tolerance to hepatocyte autoantigens and triggers T cell-mediated liver injury (31). Therefore, restoring immune tolerance balance (e.g., by targeting Treg, inhibiting aberrant antigen presentation, or modulating Teff) has become an important research direction for AIH treatment.

### 2.1 Immune signals mediated by autoantigen presentation via DCs

In AIH, DCs (especially intrahepatic dendritic cells and conventional dendritic cells) serve as key APCs by presenting liver autoantigens to initiate T cell activation, and in the pro-inflammatory microenvironment, they act synergistically with co-stimulatory signals and cytokines to promote the differentiation of naive CD4^+^ T cells into effector subsets (e.g., Th1, Th17), thereby participating in immune homeostasis imbalance and liver injury progression (32). DCs have dual functionality: on one hand, tolerogenic dendritic cells (tolDCs) can induce the differentiation of naïve CD4^+^ T cells into regulatory T cells (Tregs) by secreting IL-10 and other cytokines, thereby contributing to the maintenance of immune tolerance (33); on the other hand, aberrantly activated intrahepatic dendritic cells (HDCs) and mature conventional dendritic cells (cDCs) promote Teff polarization, exacerbate inflammatory responses, and are positively correlated with disease severity (34, 35). This imbalance between pro-inflammatory and tolerogenic functions makes DCs an important node for immune regulation and targeted therapy in AIH. Different subsets of DCs guide distinct types of CD4^+^ T cell responses through unique and partially overlapping molecular mechanisms, which largely determine the disease type and severity of AIH (34). The binding of autoantigenic peptides to MHCII triggers T cell receptor (TCR) recognition and requires the synergistic action of co-stimulatory signals, together initiating the antigen-specific activation and expansion of autoreactive CD4^+^ T cells (36); this process is often accompanied by immune tolerance imbalance (e.g., Treg dysfunction) and involvement of the inflammatory microenvironment, ultimately driving immune-mediated liver tissue damage (32). As immune sentinels distributed in the liver and lymphoid organs, DCs lose their normal immune tolerance function during the pathogenesis of AIH. After sensing liver tissue injury, they activate naive T cells passing through draining lymph nodes to initiate autoimmune responses against the liver (28, 37). Therefore, DCs are considered a key bridge connecting innate and adaptive immunity in AIH. Notably, in AIH, both DCs and T cells exhibit high heterogeneity, encompassing multiple cell subsets each with distinct developmental pathways, phenotypic characteristics, and functional localization, which provides an important perspective for understanding the disease heterogeneity of AIH (38).

### 2.2 Immune signals mediated by autoantigen presentation via Mφ

Mφ play a complex and central role in the pathogenesis of AIH, participating in both immune regulation and disease progression (39). As important immune cells in the liver, Mφ are deeply involved in the pathological process of AIH through multiple mechanisms. Their surface expresses various pattern recognition receptors such as Toll-like receptors (TLRs), particularly TLR9, as well as cytokine receptors, along with intracellular signaling molecules such as receptor-interacting protein kinase 3 (RIP3), mitogen-activated protein kinase (MAPK), JAK-STAT, PI3K/AKT, cGAS-STING, and inflammasome signaling pathways, making them key targets connecting inflammatory signals and tissue injury.

Mφ present autoantigens to naive T cells through classical mechanisms (direct presentation and cross-presentation) and alternative mechanisms (cross-dressing and MHC class II molecule dressing), leading to altered immune responses in the presence of co-stimulatory signals, ultimately resulting in liver injury and inflammation (28). Notably, Mφ can polarize into functionally distinct M1 and M2 phenotypes in response to different microenvironmental signals. M1 macrophages are primarily driven by IFN-γ and LPS via signaling pathways such as TLR4/nuclear factor κB (NF-κB), JAK-STAT1, MAPK, PI3K/AKT, and the NLRP3 inflammasome, and highly express pro-inflammatory cytokines including IL-1β, IL-6, TNF-α, and iNOS, thereby promoting inflammatory responses and tissue injury (40, 41); in contrast, M2 Mφ are induced by IL-4/IL-13 through the JAK-STAT6, TGF-β/Smads, and PPARγ pathways, and highly express anti-inflammatory factors such as arginase-1, IL-10, and TGF-β, participating in immune regulation and tissue repair (41). During the inflammatory response and liver injury in AIH, M2 macrophages are the dominant phenotype in the early immune response; depletion of resident hepatic Mφ leads to progressive worsening of liver inflammation, and M2 macrophages can effectively delay the progression of AIH. As the disease advances, they can polarize into M1 macrophages (42), manifested by significantly elevated expression of TNFα, iNOS, and IL-1β (43). The PI3K/AKT pathway, as an important intracellular signaling hub, not only participates in cell survival and metabolic regulation but also plays a key role in Mφ polarization and inflammatory responses (44). Recent studies have found that activation of the cGAS-STING pathway and its downstream pro-inflammatory cytokines can promote AIH progression (45). Therefore, in AIH, Mφ act as both initiators and amplifiers of inflammation, direct executors of tissue injury, and key drivers of disease chronicity and fibrosis. Specific interventions targeting different functional states of Mφ—such as regulating their polarization direction, blocking key inflammatory cytokine signals, inhibiting NF-κB activation, modulating the activity of signaling pathways like MAPK, JAK-STAT, and PI3K/AKT, interfering with crosstalk between signaling pathways, intervening in inflammasome assembly, or inhibiting their recruitment process, especially promoting the transition of Mφ from the M1 to the M2 phenotype—are becoming important targets for the development of therapeutic strategies for AIH.

### 2.3 Immune signals mediated by autoantigen presentation via B cells

In the pathogenesis of AIH, B cells exhibit multiple functional roles throughout various stages of the disease. As professional APCs, B cells can recognize and take up specific autoantigens via their B cell receptor (BCR), process them, and present them to CD4^+^ T cells through MHC class II molecules, while also providing co-stimulatory signals to promote T cell activation (46). Activated CD4^+^ T cells release CD40L-rich extracellular vesicles (EVs), and the CD40L on the surface of these vesicles interacts with CD40 on B cells, thereby promoting B cell proliferation, class switching, and differentiation (47). Therefore, the BCR signaling pathway and the CD40-CD40L pathway are considered potential intervention nodes for regulating B cell activation and plasma cell differentiation, thereby affecting autoantibody production. Notably, B cells and CD8^+^ T cells form an immune regulatory network through multiple mechanisms, in which IL-15, as a key inflammatory cytokine, plays an important role in promoting CD8^+^ T cell activation and functional maintenance (48). B cells can also directly participate in hepatocyte injury in AIH by producing specific autoantibodies (29). In AIH, activated B cells can differentiate into plasma cells and produce various autoantibodies, including anti-nuclear antibodies (ANA) and anti-smooth muscle antibodies (ASMA), which have important reference value in the diagnostic classification of AIH (29). However, approximately 20–30% of patients with type 1 AIH are negative for ANA, and about 10% of these patients are negative for all conventional autoantibodies (49, 50). There is no clear correlation between antibody titers and disease activity or the degree of liver injury, and the 2025 European Association for the Study of the Liver (EASL) guidelines no longer recommend subclassifying AIH based on autoantibody profiles. The BAFF/BLyS signaling axis, which is related to B cell survival and differentiation, has been associated with B cell homeostasis imbalance and autoantibody production in various autoimmune diseases and has become an important target in the treatment of AIH (51, 52).

Based on the above mechanisms, interventions targeting key steps in B cell activation and differentiation—such as targeting BCR-related signals, blocking CD40-CD40L co-stimulation, and modulating signaling axes that maintain B cell homeostasis including BAFF/BLyS—are considered to have potential therapeutic value and are expected to provide ideas for stratified treatment and long-term remission maintenance in AIH.

## 3 Role of T lymphocyte−mediated immune responses in AIH

### 3.1 Regulatory role of Th1/Th2/Treg immune network imbalance in the pathogenesis of AIH

CD4^+^ T cells are important organizers of adaptive immune responses and play critical roles in both the maintenance of hepatic immune homeostasis and the immunopathological processes of AIH (10, 53). Liver inflammation and tissue injury in AIH are associated with enhanced CD4^+^ T cell-mediated immune responses; however, the composition and functional status of CD4^+^ T cell subsets exhibit certain heterogeneity among different patients, disease stages, and treatment states (54). Alterations in the proportion and functional status of CD4^+^ T cell subsets, as well as the interactions between subsets, collectively shape the hepatic inflammatory microenvironment in AIH and are associated with persistent liver injury, although the applicability of specific pathways across different populations still requires validation by more clinical and translational studies. Studies have found that changes in the proportion of helper T cell subsets play an important role in the pathogenesis of AIH (55). Th1 cells, by secreting pro-inflammatory cytokines mainly IFN-γ and IL-2, activate CD8^+^ cytotoxic T cells (CTLs), enhance HLA molecule expression on hepatocytes, thereby amplifying the hepatic inflammatory cascade and driving cell-mediated tissue injury (32); whereas Th2 cells mainly secrete cytokines such as IL-4, IL-10, and IL-13, participate in humoral immune responses by promoting B cell differentiation into plasma cells and antibody production (56). Under healthy conditions, Th1 and Th2 cells are in dynamic balance, jointly maintaining immune homeostasis (57). However, in AIH patients and hepatitis models, a trend of enhanced Th1-like immune responses and decreased Th2 and Treg activity has been observed (58). Differentiation of Th1 cells depends on activation of the transcription factor T-bet (Tbx21) (59). In addition to promoting Th1 effector differentiation, T-bet also acts as a repressor in differentiated mature Th1 cells, preferentially inhibiting genes and pathways typically activated by type I IFN, thereby preventing abnormal amplification of the type I IFN signaling circuit triggered by the transcriptional response to its own secreted products (60). Tregs mainly secrete TGF-β and IL-10 as their primary inhibitory cytokines, among which TGF-β maintains forkhead box protein 3 (FOXP3) expression and participates in Treg differentiation through the TGF-β/Smad pathway, while IL-10 can inhibit multiple cytokines including IL-2, IFN-γ, and GM-CSF (61). Collectively, the immune imbalance in AIH involves abnormalities at multiple levels, including overactivation of Th1 responses and insufficient suppression by Th2/Treg. The Th1/Th2/Treg network constitutes a dynamic system of checks and balances in the pathogenesis of AIH.

### 3.2 Regulatory role of Th17/Treg balance in the pathogenesis of AIH

Under physiological conditions, the balance of the Th17/Treg axis maintains immune homeostasis (62). Imbalance in the Th17/Treg ratio is considered one of the key factors exacerbating immune dysregulation in AIH. TGF-β is required for inducing FOXP3 expression in naive T cells and for Treg development; Tregs exert immunosuppressive functions, including through the secretion of inhibitory cytokines, helping to maintain peripheral immune tolerance (63). In the inflammatory microenvironment of AIH, elevated levels of cytokines such as IL-6 disrupt the Treg/Th17 balance, shifting it toward pro-inflammatory Th17 cells; whereas inhibiting IL-6 expression reverses this process, restoring the balance toward protective Tregs (64). IL-6 acts synergistically with TGF-β to drive Th17 cell differentiation by activating the signal transducer and activator of transcription 3 (STAT3) signaling pathway, which interacts with two distinct conserved non-coding sequences, CNS9 and CNS6, within the Rorc locus to co-regulate the expression of RORγt (65). Th17 cell differentiation begins with the combined induction by TGF-β and IL-6, and their subsequent survival, expansion, and pathogenicity are highly dependent on IL-23 signaling. During this process, STAT3 serves as a core signal transduction molecule, being phosphorylated upon stimulation by cytokines such as IL-6 and IL-23, then translocating to the nucleus where it synergizes with the key Th17 transcription factor RORγt to promote the transcription of cytokines including IL-17, IL-21, and IL-22 (66). In contrast, Tregs express multiple inhibitory receptors such as cytotoxic T lymphocyte-associated antigen 4 (CTLA-4), glucocorticoid-induced TNF receptor-related protein (GITR), and lymphocyte activation gene 3 (LAG-3) through their master transcription factor FOXP3, thereby exerting immunosuppressive functions (67–70). In AIH patients, Treg numbers and functions are frequently reported to be defective, including decreased FOXP3 expression levels (71). Basic research has also found that the deubiquitinating enzyme Usp22 can maintain Treg function by stabilizing the FOXP3 protein, providing a new potential mechanism for understanding Treg functional instability in AIH (72).

**Figure 1:**
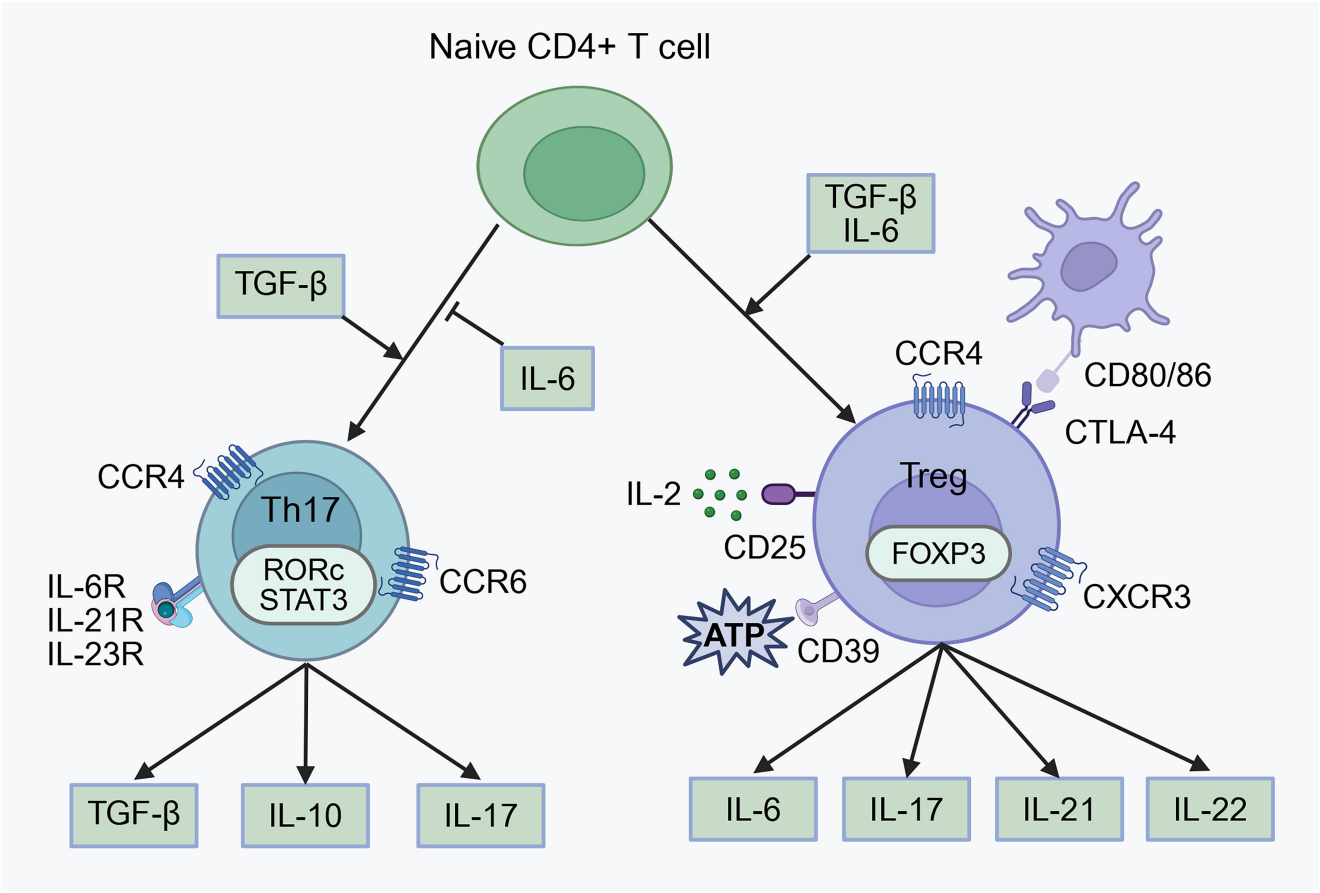
Regulatory mechanisms of the Th17/Treg balance and its imbalance in AIH. Naive CD4^+^ T cells differentiate into Th17 or Treg subsets under the induction of cytokines such as TGF-β and IL-6; through transcription factor regulation, cytokine secretion, and surface molecule interactions, these two subsets jointly maintain immune balance, and their imbalance is a key link in the pathogenesis of AIH. RORc, retinoic acid receptor-related orphan receptor C; CCR4/CCR6, CC chemokine receptor 4/6; CXCR3, CXC chemokine receptor 3; IL-6R/IL-21R/IL-23R, interleukin-6/21/23 receptors.

In summary, Th17/Treg axis imbalance participates in the pathogenesis and progression of AIH through complex cellular and molecular networks. Th17 differentiation driven by STAT3 and RORγt, along with their effector molecules, plays an important role in promoting liver inflammation and fibrosis. Meanwhile, Treg functional defects characterized by reduced FOXP3 expression or stability break immune tolerance. The mutual antagonism between RORγt and FOXP3 is key to determining the tilt of this balance (**Figure 1**).

### 3.3 Immunoregulatory role of T follicular helper cells in AIH

**Figure 2:**
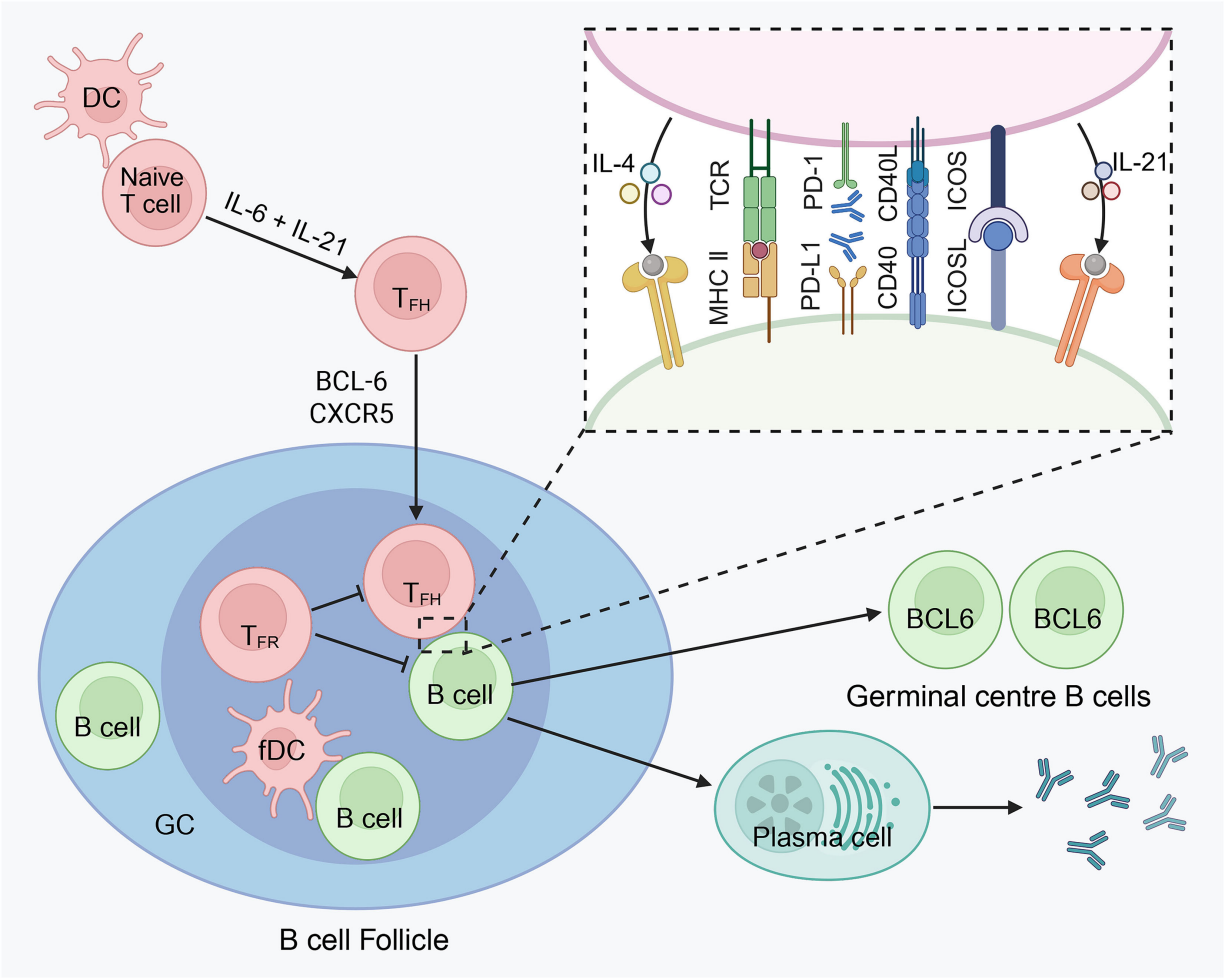
The TFH-TFR-B cell axis plays an important role in the pathogenesis of AIH. IL 6 and IL 21 secreted by dendritic cells (DC) can induce naive CD4^+^ T cells to differentiate toward the TFH lineage, accompanied by expression of CXCR5 and BCL 6. Within the germinal center (GC), TFH cells interact with B cells through multiple co-stimulatory signals, including the TCR-antigen peptide/MHCII complex, CD40L-CD40, and ICOS-ICOSL, while also secreting IL-4 and IL-21, thereby promoting B cell proliferation and eventual differentiation into plasma cells. During this process, TFR cells exert a negative regulatory role, inhibiting the functions of TFH cells and B cells to maintain homeostasis of the GC reaction. In AIH patients, the number of TFH cells is significantly increased, while the number of TFR cells is significantly decreased; the TFH/TFR imbalance is closely associated with excessive autoantibody production and disruption of immune homeostasis, thereby participating in the immunopathological process of AIH. TFH, T follicular helper cells; MHCII, major histocompatibility complex class II; ICOS, inducible T cell co-stimulator; fDC, follicular dendritic cell.

In the pathogenesis of AIH, T follicular helper cells (TFH) play an important role in connecting cellular immunity and humoral immunity, and their dysfunction is closely related to the chronicity of AIH and hepatocyte injury. As a CD4^+^ T cell subset expressing IL-21, the number of TFH is significantly increased in the peripheral blood of AIH patients, and the expression of their related factors B cell lymphoma 6 protein (BCL-6) and CXC chemokine receptor 5 (CXCR5) in peripheral blood mononuclear cells of AIH patients is also significantly higher than that in healthy controls (73). By expressing CD40L and secreting IL-21, TFH can promote B cell proliferation in germinal centers, differentiation into plasma cells, and autoantibody production. A reduction in their number and an imbalance in the TFR/TFH ratio cause this process to become uncontrolled, leading to massive autoantibody production and disruption of immune homeostasis (74, 75). Differentiation of TFH is precisely regulated by multiple signaling pathways, among which the IL-6/STAT3 signaling pathway promotes their differentiation by upregulating BCL-6 expression, whereas the IL-2/STAT5 signaling pathway negatively regulates TFH differentiation by inducing inhibitory factors such as Blimp-1 (76, 77). Elevated serum IL-6 levels and impaired immune regulation are considered important factors leading to abnormal TFH differentiation (78, 79). Hyperfunction of TFH can promote germinal center B cell activation, break the body’s tolerance to autoreactive B cells, and lead to the production of large amounts of autoantibodies (e.g., ANA, SMA, etc.) (80). Th17 and TFH form a mutually reinforcing positive feedback network through the shared cytokine IL-21: IL-21 is mainly secreted by Th17 and TFH, and not only promotes the proliferation and development of TFH and Th17 themselves but also mediates B cell differentiation into plasma cells to produce autoantibodies, thereby exerting synergistic pathogenic effects in the immunopathological process of AIH (81) (**Figure 2**).

### 3.4 CD8+ T cell-mediated autoimmune response

In the immunopathological mechanism of AIH, specific CD8^+^ tissue-resident memory T cell subsets are considered key effector populations mediating hepatocyte injury (9). Studies have indicated that in AIH, CD8^+^ T cells directly mediate hepatocyte injury through multiple mechanisms: on one hand, specific pathogenic subsets (e.g., tissue-resident memory T cells and PD-1^+^ CD8^+^ T cells) are significantly expanded in the liver and can kill hepatocytes by secreting granzymes, perforin-1, and inducing pyroptosis (82); on the other hand, CD8^+^ T cells can also actively penetrate hepatocytes via the CD44/p-ERM/F-actin pathway, a feature termed ‘emperipolesis’, which is one of the histopathological features for the diagnosis of AIH (83). Therefore, CD8^+^ T cell hyperfunction is an important driver of liver immune injury and persistent disease progression in AIH. Activation of CD8^+^ T cells does not absolutely depend on conventional cDC1 cells or the WDFY4-dependent cross-presentation pathway, but instead redundantly utilizes the functions of cDC1 and cDC2, as well as the “cross-dressing” pathway in which cDCs directly acquire peptide-MHC-I complexes from non-hematopoietic cells, with type I interferon playing a critical role in this process (84). In addition to classical regulatory pathways, emerging research provides new perspectives for understanding CD8^+^ T cell dysfunction in AIH. For example, recent studies have found that TOX expression in γδ T cells (γδ T) is downregulated in AIH patients and negatively correlated with AIH diagnostic scores. Moreover, TOX maintains the balance of γδ T development within the thymus by interacting with TCF1; its deletion leads to aberrant differentiation of pathogenic IL-17A-secreting γδ T cells (Tγδ17) and induces fatal AIH (85). These results suggest a multifaceted role for TOX in AIH, participating both in thymic development regulation and in influencing the pathogenic potential of peripheral autoreactive T cells. In a mouse model of AIH, intrahepatically expanded pathogenic PD-1^+^CD8^+^ T cells, through high expression of granzyme B and perforin-1, induce GSDMD-mediated pyroptosis of hepatocytes, thereby exacerbating liver injury and inflammation; treatment with a granzyme inhibitor blocks this process, indicating that granzyme B is a key effector molecule for the cytotoxic function of PD-1^+^CD8^+^ T cells (82). In addition to direct cytotoxicity, CD8^+^ T cells also mediate hepatocyte apoptosis via the Fas/FasL pathway, and by secreting pro-inflammatory cytokines such as IFN-γ and TNF-α, establish a complex inflammatory network within the liver, recruiting other immune cells to the site of injury (86). TNF-α, as a pleiotropic cytokine, exhibits dual functions in the progression of liver disease, promoting liver regeneration on one hand, while also participating in the development of liver fibrosis through mechanisms such as activating HSCs (87). These newly discovered mechanisms in basic immunology point to potential research directions for exploring the deeper causes of CD8^+^ T cell dysfunction in AIH.

**Figure 3:**
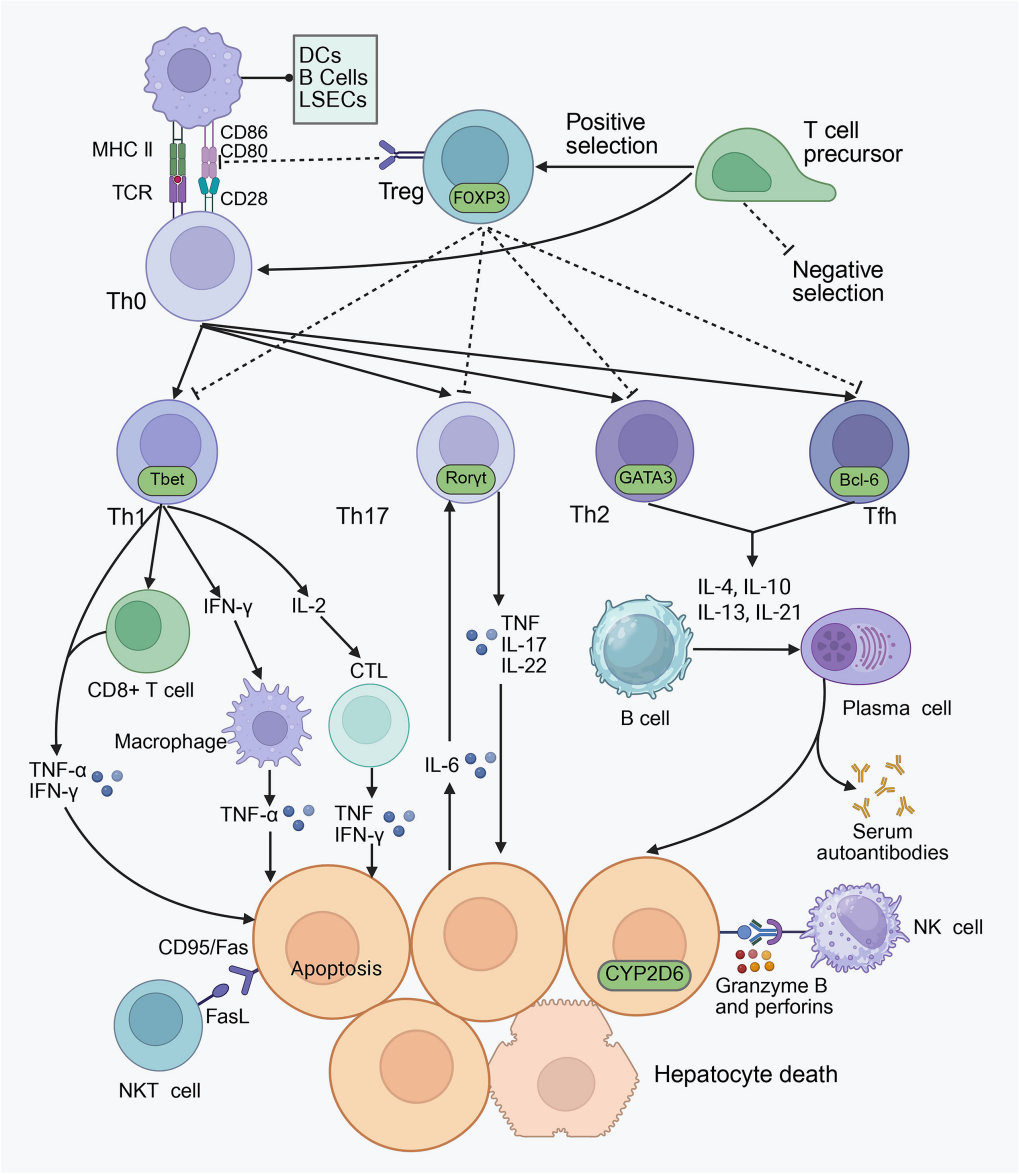
Key immune cells and their interaction network in AIH. Apoptosis is the programmed hepatocyte death mediated by granzyme B/perforins, Fas/FasL, and TNF-α as shown in the figure; hepatocyte death includes apoptosis and other forms (e.g., necrosis, pyroptosis), with the latter often accompanied by inflammation. DCs, Mφ, B cells, etc., activate T cell subsets. Overactivation of Teff and functional imbalance of Treg mediate liver injury, together constituting the immune regulatory network in AIH. GATA3, GATA binding protein 3.

In summary, CD8^+^ T cells in the pathological process of AIH integrate multiple pathways from TCR-mediated antigen recognition and immune checkpoint-mediated functional regulation to the execution of hepatocyte killing through cytotoxic granules and inflammatory cytokine networks. These studies undoubtedly provide important clues for a deeper understanding of the functional plasticity of CD8^+^ T cells and their role in chronic liver inflammation, and offer new insights for the development of more specific immunomodulatory strategies (**Figure 3**).

## 4 Role of intercellular communication in liver tissue in the pathological mechanism of AIH

As a core organ with both metabolic and immune regulatory functions, the maintenance of physiological homeostasis in the liver is highly dependent on precise and orderly intercellular communication between intrahepatic immune cells and non-immune cells. In the pathological process of AIH, the imbalance of this intricate dialogue between intrahepatic immune cells and non-immune cells is widely considered a core feature of disease onset and progression. Aberrant intercellular signal transmission participates in or accompanies multiple key steps, including the breakdown of autoimmune tolerance, amplification of inflammatory cascades, hepatocyte injury, and progression of liver fibrosis. The construction of this pathological network encompasses several levels: first, cytokines, as soluble messengers, transmit pro-inflammatory or anti-inflammatory signals between different cells, and their imbalance is central to the inflammatory cascade (88); second, direct interactions of cell surface molecules, such as the expression of adhesion molecules, are considered to provide a physical basis for immune cell migration, recruitment, and execution of effector functions (89); third, hepatocytes, as targets of immune attack, are not passive victims; they can act as unconventional APCs by taking up, processing, and presenting autoantigens on MHC molecules, thereby actively participating in and amplifying local immune responses (28). In-depth analysis of the molecular mechanisms and regulatory networks of intrahepatic intercellular communication in AIH will not only further elucidate the pathogenesis of the disease but also provide new theoretical bases and target directions for overcoming the limitations of traditional non-specific immunosuppressive therapy and developing precise, targeted novel treatment strategies.

### 4.1 Cytokine-mediated disruption of intrahepatic intercellular signaling promotes the occurrence and development of AIH

The cytokine signaling network in the liver is co-constructed by various immune and non-immune cells, including mononuclear phagocytes, lymphocytes, as well as hepatic parenchymal and non-parenchymal cells (88, 90). Under physiological homeostasis, through multiple complex regulatory mechanisms, the liver dynamically maintains a tolerogenic microenvironment characterized by anti-inflammatory and immunosuppressive features in the context of continuous exposure to gut-derived antigens and microbial products, thereby preventing excessive immune responses to harmless antigens and preserving tissue homeostasis (91). However, this homeostasis is disrupted during the pathogenesis of AIH. AIH involves functional abnormalities of CD4^+^ T cell subsets (e.g., Th1, Th2, Th17, Treg), which secrete different cytokines (e.g., IFN-γ, IL-17, IL-1β, etc.) and constitute a complex, mutually regulated signaling network involved in inflammatory regulation, exhibiting different patterns of quantitative changes between active and remission phases of AIH (92). Among these, IFN-γ is mainly produced by various immune cells including CD8^+^ T cells, CD4^+^ Th1 T cells, natural killer cells, NKT cells, and type 1 innate lymphoid cells (93). Blocking the IFN-γ signaling pathway is an effective strategy for ameliorating AIH-like liver pathological injury (94). TNF not only licenses self-aggressive CD8^+^ T cells to fully execute their cytotoxic program but also upregulates adhesion molecule expression on hepatocytes, making them more susceptible to immune cell attack (95). Activation of IL-1β is closely associated with the NLRP3 inflammasome; activated IL-1β can induce the release of other inflammatory factors by activating signaling pathways such as NF-κB and MAPK, thereby amplifying the inflammatory cascade (96, 97). IL-6 promotes pro-inflammatory responses and exacerbates hepatocyte injury and inflammatory progression in AIH by promoting the aberrant activation and differentiation of CD4^+^ T cells (especially the Th17 subset), suppressing Treg function, and disrupting the Th17/Treg immune balance (98). IL-17 plays an important role in the pathological process of AIH by driving liver inflammation, inducing immune cell infiltration, and fibrosis (99). IL-12 drives the differentiation of naive CD4^+^ T cells into Th1 cells via the STAT4-T-bet signaling pathway (100); as an upstream regulatory cytokine, IL-23 drives inflammatory responses by stimulating the differentiation and proliferation of downstream effector cells (e.g., Th17 cells); the IL-23/Th17 axis constituted by IL-23 and Th17 cells has been demonstrated to be involved in the pathogenesis of various autoimmune diseases (101). The above pro-inflammatory cytokine network achieves self-maintenance and sustained amplification of inflammatory signals through multi-level positive feedback loops. In contrast to the pro-inflammatory network, the anti-inflammatory cytokine network undertakes the function of limiting tissue damage. IL-10 is an important inhibitory cytokine in this context, effectively suppressing the production of pro-inflammatory factors by Mφ and DCs and enhancing Treg function (102). In addition, the expression levels of both subunits (EBI3 and p35) of IL-35 are significantly elevated in liver tissue and are positively correlated with the degree of liver inflammation and fibrosis; myeloid-derived suppressor cells (MDSCs) have been identified as target cells of IL-35, and IL-35 can significantly expand MDSCs and promote their production of nitric oxide (NO), suggesting that IL-35 may play an important role in the immune microenvironment of AIH by regulating MDSCs (103).

During the pathogenesis of AIH, the systemic imbalance between the pro-inflammatory cytokine network and the anti-inflammatory cytokine network leads to sustained disruption of hepatic immune tolerance, forming a vicious cycle of “enhanced pro-inflammation – weakened anti-inflammation”. Understanding the dynamic balance mechanism of this cytokine network provides a theoretical basis for the development of precision therapies targeting specific signaling pathways (e.g., targeting the IL-12/IL-23 pathway or IL-1β signaling).

### 4.2 Role of cell surface molecule-mediated intrahepatic immune signaling in AIH

In the pathogenesis of AIH, the interactions of cell surface molecules constitute a sophisticated immune regulatory network—from antigen presentation to the transmission of co-stimulatory signals, to cell migration and localization mediated by adhesion molecules—all of which are deeply involved in the development of AIH. For example, dysregulation of CD80/CD86 co-stimulatory molecules, abnormal activation of the activated leukocyte cell adhesion molecule (ALCAM)-CD6 adhesion axis, and global disruption of cell-cell interactions are widely considered core mechanisms driving disease onset and progression (104–106). Professional APCs present processed hepatic autoantigen peptides to CD4^+^ T cells via their surface MHC class II molecules, and the specific recognition of the TCR with the MHC-peptide complex constitutes the first signal for immune activation, a process considered a key step driving the occurrence of AIH (31). Meanwhile, the expression of CD80 and CD86 on B cells and monocytes is maintained or increased, and although the frequency of intrahepatic Tregs is increased and their transendocytosis function remains intact, their regulatory capacity is still insufficient to cope with the overexpressed CD80/CD86, leading to an imbalance in CD28 co-stimulatory signaling and consequently a persistently activated phenotype of conventional CD4^+^ T cells (104). In the absence of appropriate co-stimulatory signals or in the presence of aberrant co-inhibitory signals, immune tolerance may be disrupted. For example, CTLA-4, as a homologous competitive receptor of CD28, binds to B7 molecules with high affinity to deliver inhibitory signals that limit excessive T cell activation; polymorphisms or functional defects in the CTLA-4 gene may weaken this negative regulatory mechanism, thereby increasing susceptibility to AIH (107). This mechanism is not unique to AIH — the same pathological basis exists in systemic lupus erythematosus (SLE): multiple meta-analyses have confirmed that CTLA-4 gene polymorphisms are significantly associated with SLE susceptibility, particularly in Asian populations (108); functional studies have further revealed that although CTLA-4 expression is normal or even elevated in T cells from SLE patients, it is excluded from lipid rafts and fails to downregulate TCR signaling, leading to T cell hyperactivation (109). Collectively, these lines of evidence from different autoimmune diseases demonstrate that functional abnormalities of the CTLA-4 gene represent a shared susceptibility mechanism across multiple autoimmune diseases (including AIH) and contribute to the breakdown of immune tolerance. In addition, immune checkpoint molecules also play critical roles in maintaining hepatic immune homeostasis; their abnormal expression or dysfunction can lead to Treg dysfunction, Teff overactivation, and loss of immune tolerance, thereby promoting liver injury (110, 111). Beyond co-stimulatory/co-inhibitory pathways, cell-cell contacts mediated by cell adhesion molecules also play important roles in the pathological process of AIH. For instance, the interaction between CD6 and its ligand ALCAM promotes the accumulation and activation of pathogenic T cells within the liver of AIH patients (105). Similarly, the binding of MAdCAM-1 to its integrin receptor α4β7 plays an important role in mediating lymphocyte homing to the liver, and deletion of MAdCAM-1 or β7 integrin significantly reduces the severity of liver injury (89). Notably, BAFF and APRIL, as core factors of the B cell activation system, are not only essential for B cell survival and function but also co-stimulate T cells (112). Thus, multiple cell surface molecules in AIH (such as MHC, co-stimulatory/co-inhibitory receptors, adhesion molecules, and cytokine receptors)—through a complex intercellular communication network—coordinate the activation, migration, localization, and effector functions of immune cells. Once the balance of these molecular interactions is disrupted, the original immune tolerance state of the liver is broken, triggering autoimmune attacks against hepatocytes, ultimately leading to chronic hepatitis, fibrosis, and even liver failure.

### 4.3 Relationship between signal communication between hepatocytes and immune cells and the occurrence and development of AIH

Bidirectional communication signals between hepatocytes and immune cells participate in multiple stages of the pathological process of AIH. In the early stage of AIH pathogenesis, LSECs can secrete chemokines such as CXCL9 and CXCL10 to establish chemical gradients that induce T cell homing (113). Subsequently, immune cells bind to adhesion molecules ICAM-1 on the surface of LSECs via integrins (e.g., LFA-1), mediating their adhesion and crossing of the endothelial barrier to enter liver tissue; dysfunction of this process may promote the occurrence and development of AIH (106). During the active phase of AIH, the expression of these chemokines and adhesion molecules is significantly upregulated, promoting the recruitment, migration, and retention of immune cells to the liver, thereby driving and sustaining the inflammatory response (105, 114). Under normal physiological conditions, the liver, through its specialized non-parenchymal cells (including LSECs, KCs, HSCs, and DCs), cooperatively forms an inhibitory microenvironment. These cells mediate immune tolerance against self- and gut-derived antigens by producing anti-inflammatory cytokines such as IL-10 and TGF-β, as well as expressing inhibitory T cell co-stimulatory molecules such as PD-L1 (115). However, in the inflammatory microenvironment of AIH, hepatocytes abnormally upregulate MHC class II molecule expression, acquiring the function of non-professional APCs, and subsequently form MHC II-CD4 immune synapses with CD4^+^ T cells, directly presenting autoantigens to CD4^+^ T cells and participating in liver autoimmune injury (116). Meanwhile, necrotic hepatocytes release various damage-associated molecular patterns, including mitochondrial DNA, which can act as agonists of Toll-like receptor 9, activating innate immune responses and inducing neutrophil infiltration, thereby further amplifying hepatocyte injury and contributing to the progression of liver inflammation (117). In addition, CD8^+^ T cells not only induce hepatocyte apoptosis via the Fas/FasL pathway (118) but also, through the pathogenic PD-1^+^CD8^+^ T cell subset that highly expresses granzyme B and perforin-1, induce GSDMD-mediated hepatocyte pyroptosis (82), and have also been shown to actively penetrate hepatocytes via the CD44/p-ERM/F-actin pathway to form “pseudo-invasion” structures (83). At the immune regulation level, Tregs in AIH patients often exhibit reduced numbers and/or functional defects, leading to decreased suppression of Teff and sustained amplification of autoimmune responses (119). Furthermore, the interaction between hepatocytes and immune cells also involves aberrant activation of multiple key signaling pathways and molecular axes. For example, in AIH patients and ConA-induced mouse models, PGAM5 expression in hepatocytes is significantly elevated, promoting hepatocyte necrosis by activating Drp1-mediated mitochondrial fission, thereby exacerbating immune-mediated liver injury; inhibition of the PGAM5-Drp1 axis effectively reduces liver inflammation and cell death, suggesting an important role for this pathway in hepatocyte-immune system crosstalk (120). KCs, as the main resident Mφ in the liver, exhibit a dual role in AIH: on one hand, their normal function helps maintain hepatic immune tolerance; on the other hand, under inflammatory conditions, bone marrow-derived monocyte-derived Mφ are aberrantly recruited and polarized into a pro-inflammatory phenotype, releasing large amounts of cytokines such as TNF-α and IL-1β, activating the NLRP3 inflammasome, promoting pyroptosis and reactive oxygen species (ROS) production, further damaging hepatocytes (96, 121).

### 4.4 Communication between cholangiocytes and immune cells

Although AIH has traditionally been considered to primarily affect the liver parenchyma rather than the biliary system, an increasing number of studies indicate that cholangiocytes are not merely passive target cells but active participants in immune regulation and inflammatory responses. First, the interaction between cholangiocytes and immune cells is achieved through multiple molecular mechanisms. For example, lymphocytes migrate to and reside in the liver under the regulation of chemokine receptors and adhesion molecules, making close contact with hepatocytes and cholangiocytes (106). Furthermore, cholangiocytes may influence immune cell function by modulating the local microenvironment. For instance, in AIH patients and animal models, fibroblasts, HSCs, endothelial cells, and cholangiocytes in the portal area all exhibit elevated levels of junctional adhesion molecule C (JAM-C); in mouse models, blocking JAM-C or using JAM-B deficient mice significantly alleviates liver fibrosis, suggesting that the JAM-B/JAM-C pathway plays a key role in the process of fibrosis mediated by communication between cholangiocytes and immune/stromal cells (122). Notably, aberrant communication between cholangiocytes and immune cells not only drives inflammation but may also disrupt hepatic immune tolerance. Finally, communication between cholangiocytes and immune cells may also be influenced by the gut-liver axis. Studies have found that AIH patients exhibit intestinal barrier dysfunction, manifested by significantly increased intestinal permeability, leading to increased bacterial translocation to the liver, which in turn activates the RIP3 signaling pathway in hepatic Mφ, amplifying the innate immune inflammatory response in the liver; broad-spectrum antibiotic intervention significantly alleviates this process, and both RIP3 activation and liver injury are markedly reduced after intestinal sterilization, suggesting that intrahepatic immune responses are regulated by the gut microbiota, and that intestinal microorganisms participate in AIH pathogenesis via the gut-liver axis (123). In addition to barrier dysfunction, the composition of the gut microbiota in AIH patients is also significantly altered, with enrichment of opportunistic pathogenic genera such as *Veillonella*, *Lactobacillus*, and *Oscillospira*; the abundance changes of these genera have good diagnostic value (124, 125). At the level of microbial metabolites, levels of short-chain fatty acids (SCFAs) and secondary bile acids are reduced in AIH patients, while levels of lipopolysaccharide (LPS) and branched-chain amino acids are elevated (126). SCFAs and secondary bile acids can regulate hepatic immune homeostasis by activating G protein-coupled receptors (GPRs) and the farnesoid X receptor (FXR); LPS activates hepatic Mφ via the TLR4/NF-κB pathway, promoting a pro-inflammatory cytokine cascade; microbiota-derived tryptophan metabolites, specific amino acids, and others also interact with immune cells, synergistically promoting the inflammatory response in AIH (127).

**Figure 4:**
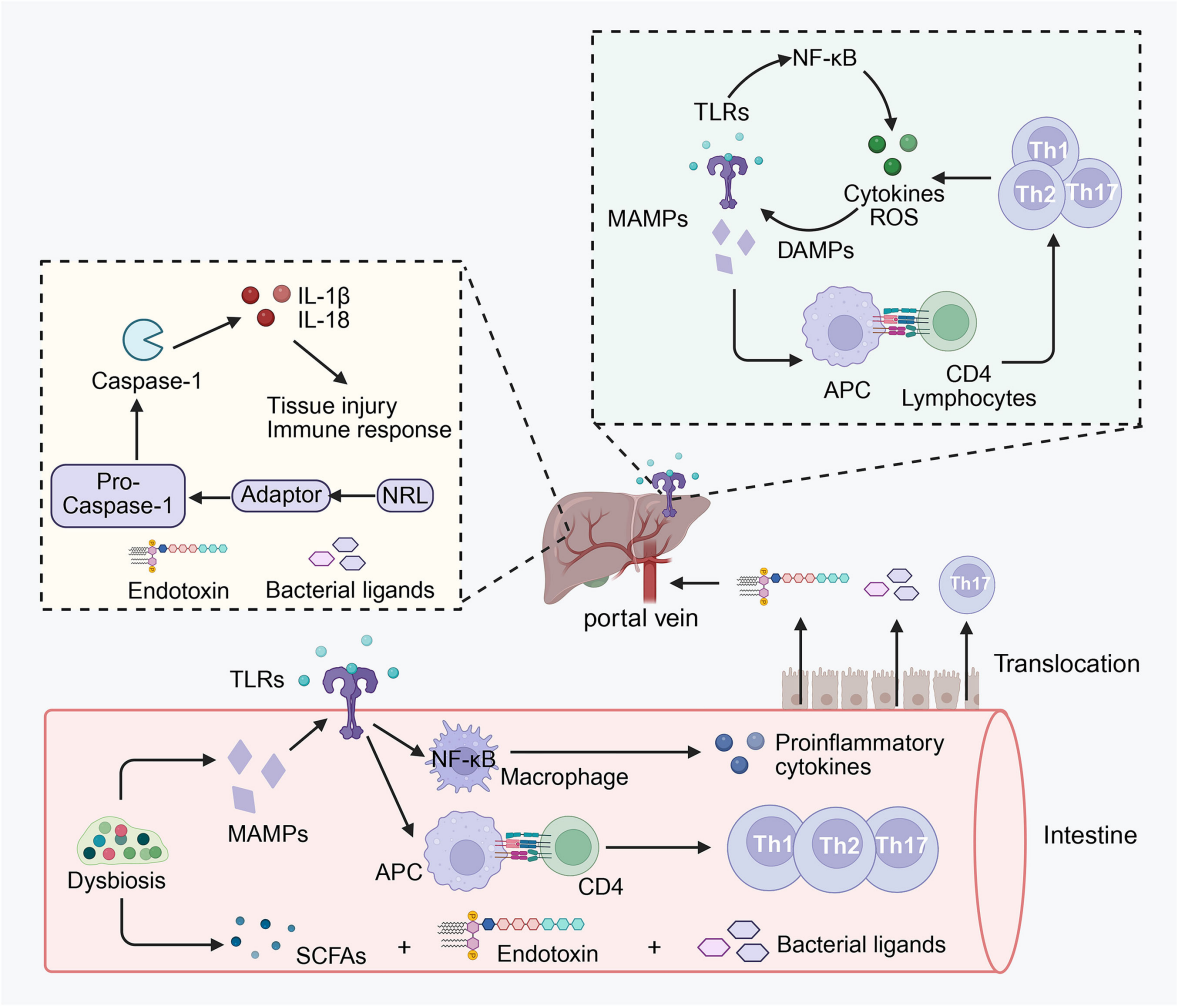
Mechanism of the gut microbiota-gut-liver axis regulating the immune response in AIH. Dysbiosis of the gut microbiota produces microbe-associated molecular patterns (MAMPs), endotoxins, etc., activates the intestinal TLRs-NF-κB pathway, induces Mφ to release pro-inflammatory factors, and promotes the differentiation of CD4^+^ T cells into Th1/Th2/Th17 via APCs. Impaired intestinal barrier allows these products to translocate into the liver through the portal vein, where they activate intrahepatic TLRs and NOD-like receptor (NLR) inflammasomes to form a pro-inflammatory amplification loop: on one hand, producing damage-associated molecular patterns (DAMPs) and ROS, exacerbating hepatocyte injury; on the other hand, sensitizing CD4^+^ T cells through molecular mimicry mechanisms, amplifying autoimmune responses, and ultimately promoting the occurrence and development of AIH. SCFAs, short-chain fatty acids.

Therefore, cholangiocytes are not bystanders in AIH; rather, they engage in complex and dynamic communication with immune cells through multiple means including cell contact, adhesion molecules, metabolites, and signaling pathways, collectively driving immune intolerance, persistent inflammation, and liver injury (**Figure 4**).

## 5 Current status of application and development of therapeutic drugs for AIH

Currently, the clinical treatment of AIH still uses glucocorticoids combined with azathioprine as the standard first-line regimen. Although this regimen can achieve remission in 80% of patients, its widespread application has always been accompanied by significant treatment challenges (128). While both drugs have clear efficacy, their non-specific immunosuppression is also associated with significant side effects. Glucocorticoids significantly increase the risks of fractures, diabetes, etc., while azathioprine may cause bone marrow suppression and hepatotoxicity (129, 130). For special populations intolerant to glucocorticoids, budesonide, due to its high first-pass effect, has become an alternative choice, but its applicability is strictly limited to non-cirrhotic patients (131); for those intolerant to azathioprine, although 6-mercaptopurine can provide an approximately 70% response rate, its tolerability remains suboptimal (132, 133); mycophenolate mofetil (MMF), as a more expensive alternative, has been shown to effectively suppress disease activity in the majority of AIH patients intolerant to azathioprine; notably, MMF is contraindicated during pregnancy (133). The core limitations common to these traditional immunosuppressants—broad targets, significant long-term toxicity, and high relapse rates after drug withdrawal—profoundly reveal the unmet clinical needs in the field of AIH treatment, thereby continuously driving researchers to explore innovative therapeutic strategies that are more targeted and aim to re-establish immune tolerance rather than broad-spectrum suppression.

Developing more specific and precise novel immunotherapeutic strategies is an important direction for current AIH drug discovery. In recent years, drug development for AIH has formed a multi-level strategic system, the core of which lies in precisely intervening at various stages of the autoimmune response. In modern therapeutic strategies for AIH, researchers are actively exploring multiple targeted therapeutic mechanisms, such as achieving B cell depletion via anti-CD20 monoclonal antibody (rituximab) and anti-BAFF antibody (belimumab) (134); blocking key inflammatory cytokines such as TNF-α via anti-TNF-α monoclonal antibody (infliximab) (95); modulating T cell activation through standard mechanisms such as blocking co-stimulatory signals between T cells and APCs (135); as well as emerging approaches such as intervening in intracellular key signaling pathways (e.g., targeting the MCP-1/p38 axis) to inhibit excessive activation of immune cells (136) and modulating metabolic-immune crosstalk, such as targeting key metabolic checkpoints of Tregs to re-establish immune homeostasis (137), thereby dismantling pathogenic immune responses from multiple dimensions. With deeper understanding of disease mechanisms, the focus of drug discovery has shifted from mere immunosuppression to the re-establishment of immune tolerance. Small molecule inhibitors, such as JAK-STAT pathway inhibitors, can block specific cytokine signaling and precisely regulate the direction of Th cell differentiation (138). Meanwhile, emerging strategies including TLR4 antagonists targeting innate immunity (e.g., low-dose naltrexone) and Treg therapies that restore immune balance via cell infusion (e.g., adoptive Treg transfer) enrich treatment options from different dimensions (30, 139). In summary, therapeutic strategies for AIH are undergoing a critical transformation from broad-spectrum suppression to precise regulation. This deepening understanding is an important direction driving the development of next-generation innovative therapies for AIH. To systematically present the current status of drug development and application in the field of AIH treatment, we have compiled multiple classes of drugs/therapies ranging from traditional precise targeted therapies to cutting-edge cell therapies, and from immune regulation to metabolic intervention. Around the five major dimensions of “drug category – mechanism of action – clinical stage – limitations – application prospects”, we have organized the positioning and potential of different therapeutic approaches in AIH (**Table 1**).

**Table 1:** Current therapeutic agents and emerging strategies for autoimmune hepatitis.

Drug category	Specific drugs/therapies	Target/mechanism of action	Clinical application stage	Limitations	Application prospects
Biologics	Rituximab (anti-CD20 monoclonal antibody) (19)	CD20 molecule (B cell surface marker), depletes CD20+ B cells, blocks autoantibody production and antigen-presenting function	Clinical application (for refractory AIH patients who have failed or are intolerant to conventional therapy, based on case observation data)	May increase risk of infection; Long-term safety requires validation in larger sample studies; Only applicable to specific populations	Provides a precise option for refractory AIH associated with abnormal B cell activation; future work needs to establish selection criteria for the population that will benefit
	Infliximab (anti-TNF-α agent) (140)	TNF-α molecule, neutralizes overexpressed TNF-α, blocks its activation of the NF-κB pathway and its pro-inflammatory cell infiltration effect	Clinical exploration (efficacious in individual case reports, lacking validation from prospective randomized controlled trials)	Potential risk of hepatotoxicity (may aggravate drug-induced liver injury); Efficacy and safety are highly controversial	If subsequent trials confirm safety, it could be used for AIH subtypes with high TNF-α expression, requiring patient selection based on biomarkers
	Abatacept (CTLA-4-Ig fusion protein) (141)	CD80/CD86 molecules on the surface of APCs, blocks their binding to CD28 on T cells, inhibits full T cell activation	Clinical exploration (preliminary clinical studies show improvement in inflammatory markers, direction of individualized therapy)	Significant variability in efficacy due to AIH heterogeneity; May impair anti-infective immunity	Provides a concept for immune tolerance reconstruction in AIH patients with T cell overactivation; future optimization of dosing regimens is needed
Small molecule inhibitors	Tofacitinib (JAK inhibitor) (138)	JAK kinase, blocks signaling of γ-chain cytokines including IL-6, IL-12, and IL-23, inhibits Th17 differentiation and pro-inflammatory cytokine release	Phase III clinical trials (efficacious in animal models; multiple Phase III studies are exploring the optimal therapeutic window)	Lack of large-scale clinical data for the AIH indication; Long-term safety (e.g., hematologic effects) needs to be clarified	High oral convenience, suitable for patients requiring long-term treatment; may become a core drug for precisely regulating cytokine pathways in the future
	Belimumab (BAFF inhibitor) (142)	BAFF (B cell activating factor), regulates B cell maturation, differentiation, and survival, reduces autoantibody production	Clinical exploration (efficacious in clinical observations, lacks evidence-based medical evidence for the AIH indication)	The status of the AIH indication has not been clearly defined; Limited efficacy in AIH subtypes that are not dominated by B cells	Suitable for AIH patients with hypergammaglobulinemia and high titers of autoantibodies; future clinical data related to the indication need to be supplemented
	Secukinumab/Ixekizumab (IL-17 inhibitors) (142)	IL-17 molecule, neutralizes IL-17 secreted by Th17, blocks neutrophil recruitment and inflammatory response	Clinical exploration (based on the pathological basis of high IL-17 expression, and experience with efficacy in other immune diseases)	Limited clinical data related to AIH; May increase the risk of mucosal infections (e.g., Candida infection)	Provides a new direction for AIH subtypes dominated by Th17 cells; future validation of the specificity of liver inflammation improvement is needed
Immuno-microenvironment modulating drugs	Obeticholic acid (FXR agonist) (143)	FXR (farnesoid X receptor), inhibits the NF-κB pathway to exert anti-inflammatory effects, and inhibits HSC activation to exert anti-fibrotic effects	Clinical research (improves biochemical parameters, delays fibrosis, multiple benefits)	Some patients may experience gastrointestinal side effects such as pruritus; The long-term effects on liver function need to be clarified	Possesses dual anti-inflammatory and anti-fibrotic effects, suitable for AIH patients with concomitant fibrosis, and may become a core component of combination therapy in the future
	JKB-122 (TLR4 inhibitor) (144)	TLR4 (Toll-like receptor 4)	Phase II clinical trial stage	The specific target and signaling pathway are not yet fully defined; The optimal dosage and treatment duration still need to be explored	Provides a promising new direction for the treatment of chronic liver diseases such as AIH, particularly in inhibiting liver inflammation and fibrosis
Cell therapy	Regulatory T cell (Treg) infusion (145)	Expanded functional Tregs, re-establish immune tolerance through competitive binding of CTLA-4 to CD80/CD86 and secretion of inhibitory cytokines	Preclinical/early clinical (efficacious in other autoimmune diseases, under exploration in the AIH field)	The stability and persistence of infused cells in the inflammatory environment need to be improved; Standardization of cell preparation is challenging	Holds promise for achieving immune tolerance reconstruction in AIH, and may become a curative treatment direction in the future; breakthroughs in cell engineering technology are needed
	Mesenchymal stem cell (MSC) therapy (146)	Paracrine secretion of immunomodulatory factors such as IL-10 and TGF-β, inhibits pathogenic T cells, promotes hepatocyte regeneration	Preclinical/early clinical (suitable for refractory patients with concomitant fibrosis, multi-mechanism action)	Standardization of cell source has not been resolved;The optimal route and timing of infusion are unclear	Provides a new option for refractory AIH patients with concomitant fibrosis; future optimization of treatment regimens is needed to enhance tissue repair effects
Metabolic/anti-fibrotic combination drugs	Pirfenidone (anti-fibrotic drug) (147)	TGF-β pathway, collagen deposition, inhibits fibroblast proliferation, blocks fibrosis progression	Clinical exploration (used in combination with immunosuppressants, targeting the fibrotic stage of AIH)	Cannot control AIH inflammation when used alone; May cause gastrointestinal side effects (e.g., nausea, diarrhea)	Provides disease-modifying therapy for the fibrotic stage of AIH; the “anti-inflammatory + anti-fibrotic” combination treatment model needs to be promoted in the future
	Probiotics/prebiotics/fecal microbiota transplantation (FMT) (148, 149)	Gut microbiota, ameliorates dysbiosis, reduces LPS translocation, inhibits innate immune responses activated by TLRs	Clinical exploration (adjuvant therapy, improves intestinal barrier, indirectly regulates immunity)	Efficacy is greatly influenced by microbiota typing; Lack of standardized intervention protocols (e.g., strain selection, dosage)	As an adjuvant therapy for AIH, it can be combined with microbiota detection in the future to achieve “precision microecological regulation”

In the past five years, preclinical studies in the field of AIH treatment have achieved a series of advances, yielding a variety of potential active compounds derived from natural products or artificially synthesized. As shown in the table below, these compounds, by regulating key targets such as c-Jun, NF-κB, and STAT3, have effectively alleviated immune-mediated liver injury in animal models induced by ConA and other agents, laying a solid foundation for the development of novel targeted drugs (**Table 2**).

**Table 2:** Representative preclinical compounds with protective effects in AIH models.

Potential active compounds	Research model	Modeling agent	Regulated targets/mechanisms	Research highlights and remarks
Naringin (150)	Mouse AIH model	ConA	Targets and binds to key targets such as ALB, AKT1, IL-1β, IL-6, and TNF, inhibits inflammatory signaling pathways, increases serum albumin levels, and alleviates liver inflammatory injury	Natural flavonoid compound. Through network pharmacology combined with molecular simulation validation, it has demonstrated significant hepatoprotective effects in in vivo experiments, with high safety
MSA (94)	Mouse AIH model	ConA	Specifically downregulates IFN-γ expression, inhibits the activation of the downstream JAK1/2-STAT1 signaling pathway, and reduces inflammatory cytokine release and hepatocyte apoptosis	Itaconic acid isomer, without the side effects of interfering with energy metabolism. The mechanism was validated in tissue specimens from AIH patients, showing prominent potential for clinical translation
Piceatannol (PIC) (151)	Mouse AIH model	ConA	By specifically binding to the transcription factor c-Jun, it inhibits the immune activity of T cells and Mφ	For the first time, it was discovered that the natural product piceatannol can effectively treat AIH by directly binding to the transcription factor c-Jun, thereby dual-inhibiting the immune activity of T cells and Mφ, and revealing the potential of c-Jun as a new therapeutic target for AIH
Glycyrrhizic acid (152)	Mouse AIH model	ConA	Alleviates ConA−induced acute liver injury by regulating the function of MoMFs	It was revealed for the first time that glycyrrhizic acid effectively alleviates immune-mediated acute liver injury by precisely regulating the recruitment and function of monocyte-derived Mφ (MoMFs), providing a new and clearer cellular and molecular mechanism explanation for its clinical treatment of related liver inflammation
Astaxanthin (153)	Mouse AIH model	ConA	ASX has the potential to improve liver injury by regulating the number and function of CD8+ T cells	Using mass cytometry and single-cell sequencing, this study first revealed that astaxanthin treats AIH by reshaping the function and transcriptome of CD8^+^ T cells, providing mechanistic support for its application and opening new directions for AIH immunology and targeted therapy
Sophoricoside (154)	Mouse AIH model	ConA	SOP significantly reduced the expression of phosphorylated p65 NF-κB and the enhancement of its nuclear translocation in the livers of AIH mice and in AML12 cells stimulated with lipopolysaccharide	This study first demonstrated that the natural compound sophoricoside protects the liver from autoimmune attack via dual antioxidant and anti-inflammatory mechanisms: it alleviates oxidative stress and directly inhibits the NF-κB pathway, making it a promising multi-target candidate for treating autoimmune liver disease
Zingerone (155)	Mouse AIH model	ConA	Zingerone exerts a protective effect in a Con A-induced acute liver injury model by inhibiting M1 macrophage polarization and suppressing the NF-κB, mitogen-activated protein kinase, and interferon regulatory factor signaling pathways	Zingerone effectively alleviates T cell-mediated acute immune-mediated liver injury by inhibiting the key NF-κB signaling pathway, thereby broadly suppressing the production of pro-inflammatory cytokines and immune cell infiltration
Succinic Acid (156)	Mouse AIH model	ConA	Succinate improves ConA-induced liver injury by regulating immune balance, inhibiting pro-inflammatory factors, and promoting anti-apoptotic proteins in the liver	For the first time, it is revealed that the endogenous metabolite succinic acid effectively alleviates immune-mediated acute liver injury through two synergistic mechanisms: remodeling the hepatic inflammatory microenvironment and directly inhibiting hepatocyte apoptosis. This provides a new theoretical basis and a potential therapeutic molecule for treating liver inflammatory diseases via a “metabolic intervention” strategy
AMDS (40)	Mouse AIH model	ConA	AMDS ameliorates ConA-induced AIH by reducing hepatic neutrophil infiltration, inhibiting M1 macrophage polarization, and antagonizing NLRP3 inflammasome activation	For the first time, garlic-derived AMDS alleviates immune liver injury by dual inhibition of M1 polarization and NLRP3 inflammasome activation, offering a new drug candidate and strategy targeting Mφ inflammatory pathways
D-mannose (157)	Mouse AIH model	ConA、α-GalCer	D-mannose treatment reduces hepatic inflammation and inhibits liver injury by increasing Tregs	It was innovatively discovered that the natural monosaccharide D-mannose effectively promotes Treg differentiation and function by reprogramming T cell metabolism, thereby significantly ameliorating AIH. This provides a new theoretical basis and a highly promising therapeutic strategy for safely and precisely inducing immune tolerance through a “metabolic intervention” approach
Gastrodin (158)	Mouse AIH model	ConA	Gastrodin pretreatment exerts a protective effect against ConA-induced acute hepatitis by inhibiting the IL6/JAK2/STAT3 pathway	This study first shows that Gastrodin ameliorates immune-mediated acute liver injury by inhibiting the IL-6/JAK2/STAT3 pathway, opening new applications and confirming this pathway as a key therapeutic target
3-O-acetyloleanolic acid (159)	Mouse AIH model	ConA	3-O-acetyloleanolic acid shows great potential as a candidate therapeutic agent for alleviating liver injury in AIH by directly targeting and activating the FXR pathway	This study found that the oleanolic acid derivative 3-O-acetyloleanolic acid alleviates AIH by directly activating FXR, thereby co-regulating bile acid metabolism and immune-inflammatory pathways, offering a novel metabolic-immune intervention strategy
zVAD (160)	Mouse AIH model	ConA	zVAD alleviates ConA-induced liver injury by increasing the sensitivity of Mφ to necroptosis through IL-10-induced TNFR1 expression	This groundbreaking study discovered that the classical apoptosis inhibitor zVAD alleviates AIH by increasing the sensitivity of Mφ to TNFR1-mediated necroptosis, thereby inducing their selective clearance, ultimately effectively relieving AIH. This proposes a novel strategy for AIH treatment by “eliminating pathogenic immune cells” rather than “suppressing their function”
Itaconate (161)	Mouse AIH model	S100 antigen	Itaconate reduces autophagy and maturation of DCs by regulating the PI3K/AKT/mTOR pathway, thereby ameliorating liver inflammation in S100-induced AIH mice	This study pioneeringly demonstrated that itaconate suppresses DC maturation and autophagy via the PI3K/AKT/mTOR pathway, thereby blocking AIH initiation at the upstream of immune responses, offering a new immunometabolic target and theoretical basis for AIH therapy
Sulforaphan (SFN) (162)	Mouse AIH model	S100 antigen	SFN significantly improves S100-induced EAH by activating the AMPK/mTOR signaling pathway, enhancing autophagic flux, and alleviating pyroptosis	This groundbreaking study found that SFN enhances protective autophagy by activating the AMPK/mTOR signaling pathway, and the enhanced autophagy effectively clears pyroptosis-inducing factors, thereby inhibiting inflammatory cell death (pyroptosis) in AIH, providing a novel strategy of “enhancing autophagy to inhibit pyroptosis” for the treatment of AIH
Nimbolide (163)	Mouse AIH model	S100 antigen	Nimbolide can effectively suppress inflammation in the livers of AIH mice and in AML12 cells by inhibiting HDAC3 expression	This groundbreaking study found that the natural product Nimbolide reprograms the inflammatory gene expression profile of intrahepatic immune cells by targeting and regulating the key epigenetic modifier HDAC3, thereby effectively alleviating AIH, and established HDAC3 as a novel epigenetic target for the treatment of AIH for the first time
Hydroxychloroquine (HCQ) (164)	Mouse AIH model	S100 antigen	HCQ acts on GRK2 translocation, inhibits metabolic-related PI3K-AKT and inflammation-related JAK2-STAT3 signaling in T lymphocytes, thereby regulating lipid metabolism for T cell function, and subsequently modulating Treg differentiation and function	This study revealed that HCQ blocks the GRK2-PI3K interaction in T cells, inhibiting key AIH pathways, thus providing a new mechanism and identifying the GRK2-PI3K interface as a promising drug target
Vitexin (165)	Mouse AIH model	S100 antigen	Vitexin improves liver injury in EAH mice by activating the AMPK/AKT/GSK-3β pathway and upregulating the Nrf2 gene	In a human-like model, Vitexin activates the AMPK/AKT/GSK-3β cascade to upregulate Nrf2-mediated antioxidant defense, protecting hepatocytes from oxidative damage and alleviating AIH

## 6 Discussion

As a chronic inflammatory liver disease characterized by complex pathological mechanisms and highly heterogeneous clinical phenotypes, AIH poses a continuous burden and clinical challenges for liver disease prevention and chronic disease management. The current treatment paradigm centered on broad-spectrum immunosuppression is increasingly revealing its shortcomings due to its inherent target non-specificity, significant adverse effects, and limitations in refractory patients (166). Over the past few decades, although research on AIH has gradually expanded from single molecular mechanisms to the cellular level, the analysis of the systemic regulatory network involving multi-cellular interactions and multi-pathway synergy within the liver microenvironment remains insufficient. Most potential therapeutic targets and compounds are still at the preclinical or early clinical exploratory stage, making it difficult to achieve effective translation from basic research to clinical application.

This study focuses on elucidating the molecular signaling mechanisms of aberrant activation of core immune cells and the regulatory principles of intercellular communication within the liver microenvironment during the pathogenesis of AIH. Centering on immune cells such as DCs, Mφ, and T/B lymphocytes, it systematically reviews their functional imbalance characteristics in the breakdown of immune tolerance and amplification of inflammatory cascades, clarifying the specific roles of different immune cell subsets in the pathogenesis of AIH. At the same time, focusing on the bidirectional dialogue between hepatocytes, cholangiocytes, and immune cells, this study analyzes the core roles of intercellular communication mechanisms—including the cytokine network, cell surface molecule interactions, and the gut-liver axis regulation—in the progression of AIH. However, current research still has many shortcomings and limitations. For example, the analysis of the heterogeneity of hepatic immune cells remains insufficient; the spatiotemporal dynamic changes, functional characteristics, and mutual regulatory networks of different cell subsets at various stages of AIH have not yet been fully elucidated. The specific molecular circuits by which metabolic reprogramming mediates immune cell dysfunction and inflammatory signaling drives liver fibrosis are still unclear.

Beyond the aforementioned scientific gaps, two additional important considerations warrant attention. First, current AIH datasets exhibit significant population and geographic bias — existing studies are predominantly derived from Caucasian populations in Europe and North America, with underrepresentation of Africa, Latin America, and parts of Asia (167–169). For instance, in a 2024 international multicenter study of 1,260 cases of immune-related hepatitis, only 0.3% (4 cases) were reported from Africa, compared with 40.8% from Europe and 34.6% from the Americas. This bias implies that current knowledge, diagnostic criteria, and treatment strategies for AIH — including the “approximately 10%-20% poor treatment response” figure cited earlier — are primarily based on Caucasian data and may not be directly generalizable to other populations. Therefore, cross-national and cross-ethnic collaborative efforts are urgently needed to establish more globally representative cohorts. Second, the female predominance in AIH represents another striking epidemiological feature, with a female-to-male ratio of 4:1 for type 1 AIH and 10:1 for type 2 AIH (170). The mechanisms potentially contributing to this sex bias involve multiple factors, including sex hormones (estrogen enhances humoral immune responses and affects dendritic cell activation), X-linked genetic factors (females carry two X chromosomes with immune-related genes such as TLR7, FOXP3, and CXCR3, and X-chromosome inactivation escape may lead to overexpression of these genes), environmental and microbiome differences, and defects in Treg-mediated peripheral immune tolerance (171–173). However, the precise molecular pathways underlying these sex differences remain incompletely elucidated and require further mechanistic validation.

Based on current findings, this study integrates the current clinical treatment regimens for AIH and the research progress of drugs at different developmental stages, sorting out the research and development system from traditional broad-spectrum immunosuppression to precision immune regulation, and from single-target intervention to multi-dimensional synergistic therapy, providing a comprehensive theoretical basis and research clues for the in-depth analysis of AIH pathological mechanisms and the development of novel therapeutic strategies.

Future research on AIH needs to combine cutting-edge technologies such as multi-omics, spatial biology, and immunometabolomics to deeply analyze the synergistic regulatory network among cells in the liver microenvironment, further elucidate its unique pathological mechanisms, and explore individualized diagnostic and therapeutic approaches, so as to achieve the transition of AIH from broad-spectrum immunosuppression to precision immune regulation, promote individualized treatment and immune tolerance reconstruction, and provide more effective theoretical support and therapeutic means for clinical diagnosis and treatment.
